# Monthly Summary

**Published:** 1897-12

**Authors:** 


### Monthly Summary.

FAULTY NUTRITION—By S. Henry Dessau, M. D.—We may rightly regard the condition of early decay of the teeth, either temporary or permanent, as one of faulty nutrition, and this should receive the attention of the specialist as well as the general practitioner. To be well informed on this point is to have at hand a remedy, for fortunately the condition of rickets and allied complaints is amenable to correction and cure. The health of the pregnant mother should be kept at the highest point by careful regard and attention to outdoor exercise, and the maintenance of all the physiological functions. Added to this, her diet should be of that combination to secure a maximum quality of nutrition. The infant requires fresh, pure air, and plenty of it. No sleeping in closed rooms full of stale, overheated and over-scented air. It should be well protected by proper and sensible dress from cold and dampness. Above all, its food should be what nature has provided for it, with due regard to the mother's condition. In the unfortunate absence of mother's milk it should have the best substitute, which is by all odds good cow's milk, properly modified by modern methods to suit its digestion. All patent foods, each advertised as the best, should be severely rejected. They are without doubt a most prolific factor in the causation of rickets and allied complaints. Milk should be the sole article of food until the eruption of the first full group of teeth, whether mother's milk or cow's milk be used and that exclusively. Unless proper judgment is shown as to the quantity given and time allowed for the stomach to become emptied before given again, trouble in the form of digestive disturbances is sure to follow. It is, in my opinion, of the highest importance that the infant should not be overfed. This, in my experience, is a pernicious practice, and the one most difficult to combat in the nursery. If we could only live on half what we do humanity, from the cradle to the grave, would be the better, brighter and broader for the doing of it. —*Cosmos.*

---

A. COLORED LADY DOCTOR.—The Louisiana State Board of Medical Examiners has granted a diploma to practice medicine to Mrs. Emma Wakefield, a lady of color. She is said to be the first woman of her race in the South, or indeed in the Union, to receive this privilege. Mrs. Wakefield is the daughter of Ex Senator Wakefield, and a graduate of the medical department of the New Orleans Colored University. We presume that this lady doctor will practice exclusively among those of her own race, as the objection among the white folk in the United States to being brought in close contact with their colored fellow citizens is very great. —*Dental Brief.*

---

FOR INFLAMED EYES.—Break in small pieces and put into a bowl four poppy heads. Pour boiling water over them and allow it to draw for ten minutes, then strain. Saturate soft linen and bathe the eyes several times a day.

---

A GOOD CEMENT.—An excellent cement for mending almost anything may be made by mixing together litharge and glycerine to the consistency of thick cream or fresh putty. The cement is useful in mending stone jars or any coarse earthenware, stopping leaks in seams of tin pans or wash-boilers, cracks and holes in iron kettles, etc. It may also be used to fasten on lamp tops, or tighten loose nuts, to secure loose bolts whose nuts are lost, to tighten loose joints of wood or iron, or in many other ways about the various kitchen utensils, the range, sink, and in the pantry fittings. In all cases the article mended should not be used till the cement has hardened, which will require from one day to a week, according to the quantity of cement used. This cement will resist the action of water, hot or cold, acids, and almost any degree of heat.—*Bottler's Gazette.*

---

A LAKE OF PETROLEUM.—While the whole world is excited over the gold discoveries in the North, sight has been lost of another discovery that promises to be of great value in the development of that section. Some months ago a lake of almost pure petroleum was discovered, and samples were sent to Seattle for analysis.

The find is reported to be of great richness. A company has been formed in Seattle to handle the product, and travelers from there say that the company intends to put in on the Alaskan market at once. The lake is of unknown depth, several miles wide and five to six miles in length, and the quality of the petroleum is said to be of the finest. The lake is only two miles from the ocean. The hills surrounding it are said to be rich in coal and asphalt. It is the expectation of the owners of the lake to take its products into the mining camps of Northern Alaska whenever the waterways will permit.—*Tribune.*

---

PREVENTION OF TARTAR.—Rinse the mouth freely once a day with water in which a pinch of alum has been dissolved. It is harmless to the teeth and keeps the gums in good condition, preventing the accumulation of tartar.—C. N. Peirce, in *International Dental Journal.*

WHAT TO TRY.—

Try cranberries for malaria.

Try a sun bath for rheumatism.

Try clam broth for a weak stomach.

Try cranberry poultice for erysipelas.

Try a wet towel to the back of the neck when sleepless.

Try swallowing saliva when troubled with sour stomach.

Try buttermilk for removal of freckles, tan and butternut stains.

Try breathing the fumes of turpentine to relieve the whooping cough.

Try taking your cod liver oil in tomato sauce if you want to make it palatable.

Try a hot, dry flannel over the seat of neuralgic pain, and renew it frequently.

Try a cloth wrung out from cold water, put about the neck at night, for sore throat.

Try walking with your hands behind you if you find yourself becoming bent forward.

Try planting sunflowers in your garden if compelled to live in a malarial neighborhood.—*Health Monthly.*

---

ENGLISH PEOPLE'S TEETH.—Education is playing sad havoc with the teeth of modern generations. So an experienced dentist in the West End says, and he ought to know. Formerly decayed teeth were generally attributed to a secret fondness for bon-bons, but the idea is, it appears, quite a mistake. In fact, sugar is rather nourishing than otherwise. The truth is that the ancient sturdy, square jaw of the English race is changing, through lessons and book learning, to an angular or V.shape, which presses the molars one upon the other, does not give them room to grow, and will in time prevent some of them cutting at all. Indeed, this catastrophe is not infrequent already. In many the original teeth are becoming less in number than they ought to be, and often the "wisdomers" fail to appear. This is an "educated jaw" is lamentable. The new facial form is, according to the dentist, much cultivated by ladies, who find that it is popular among gentlemen. The latter like women with the "educated" angle of chin, which generally carries with it pearly, but very frail teeth. If the specialist be right, this process of selection, aided by further lessons and more study, will, in the course of time, produce a race without any teeth at all. Then will the dentist make fortunes—for people will require complete artificial sets from babyhood onward.—*Telegraph.*

---

THE MICROMOTOSCOPE.—A micromotoscope has been invented, which is a kinetoscope for photographing small life in motion as seen in the microscopic field. The pictures are taken by the gelatin film at from 5,000 to 15,000 magnifications and at a rate of 16 to 2,560 per minute.

The inmates being magnified thousands of times when projected on a screen, the views of some of the families of microbes are very realistic. It has been learned that some of them possess great intelligence.

The photographs of blood in circulation in the thinnest part of the ears and of the fingers, showing its capillary and arterial motion, and the changes going on in the white cells, are of great interest. They indicate something of the nature of life and disease. The stream of circulating human blood is so swift that the eye cannot keep pace with it, and the changes in the white blood cells are correspondingly rapid. Some of the pictures show a white cell on the fast moving stream, like a white cap on the sea, constantly changing its shape. It throws out or takes in its arm like an octopus, seizing the microbes in its path. In disease this movement of its arms takes place with much less energy than in health.

These pictures cannot fail to be of great value in the study of diseases. The micromotoscope will greatly aid in the investigation of phenomena of action of ameboid life in water.—*Boston Transcript.*

POPULAR ERRORS CONCERNING SNAKES.—From time immemorial, snakes have been regarded as creations of nature fit only to be despised and trampled under foot; and the people of to-day fulfill the scriptural prophecy which says "thou art cursed above every beast of the field." Such a universal feeling of dread and distrust of snakes exists, that, as a consequence very little is known concerning them.

A popular error is that snakes can sting a person, with whom they come in contact, It is an absurd impossibility, for the tongue of a snake is a very delicate organ, used chiefly for feeling and tasting. A snake, crawling over the ground, will at intervals put out its tongue to feel or perhaps taste of the objects it is passing. Frequently have I seen the garter-snake, crawl up to a frog that had been motionless for some time, feel of it with its tongue, and then, as if it tasted good, would proceed to swallow it. It evidently tastes water or milk before drinking, for it feels or tastes either liquid, and, finding it good, begins to drink. Very often when seemingly attracted by music from a flute, the same species would raise a third of its length off the ground, gently swaying its body, and slowly wave its tongue up and down as though it were hearing with that organ.

Many persons firmly believe that snakes charm their prey. Many affirm, others deny, that the statements made are correct, and the popular press of the day, eager to please its patrons, will now and then serve up interesting accounts of serpent fascination. Thus it is that the public, constantly hearing some exaggerated snake story, will look more and more askant at snakes, killing them whenever found, and thus destroying the many non-venomous of our clime, which have been proved to be useful friends of the farmer in reducing the number of injurious insects.—Armitage Green, in *Pop. Science.*

---

REMOVAL OF BLOOD STAINS.—Rinse hands, napkins, instruments, etc., in warm water, to which a teaspoonful of tartaric acid has been added. Rinse afterwards in plain water.—*Record.*

PRESERVING GUTTA-PERCHA.—Gutta-percha for filling purposes may be preserved in good condition for years b keeping it in a solution of table salt.—*Dominion Journal.*

---

MASSAGE OF THE GUMS.—In inflammation of the gums, and other disturbed circulation about the gingivæ, massage with the ball of the finger will be found very useful. It presses the blood out of the distended capillaries, hurries the circulation in the sluggish blood-vessels, giving tone to the whole local territory, re-establishing the nutrient currents and promoting resolution in inflammation.—W. E. Barret, in *Dental Weekly.*

---

TO CEMENT METAL TO GLASS :—

| | | |
|---|---|---|
| Rosin, | - - - | 3 parts. |
| Soda, | - - - - | 1 part. |
| Water, | - - - | 5 parts. |

Boil until a soap is formed.

To fifty parts of this soap add slowly, and with constant stirring, one hundred parts plaster of Paris.—*American Druggist.*

---

IT is poor economy to use old or poor burs. We know an excellent operator who lost much of his practice in this way. Both he and his patients suffered for it. One of the wisest investments in the operating-room is a good dental engine run by a motor, and a full equipment for the front and back action.—*Dominion Dental Journal.*

